# Regular vitamin C supplementation during pregnancy reduces hospitalization: outcomes of a Ugandan rural cohort study

**DOI:** 10.4314/pamj.v5i1.56178

**Published:** 2010-05-30

**Authors:** Unim Hans, Byamukama Edward

**Affiliations:** 1 Gastroenterology Unit, Department of Clinical Sciences, University “La Sapienza”, Rome, Italy; 2 St. Mary‘s Health Center, P.O.BOX 9 Kyeibuza, Kiruhura District , Uganda

**Keywords:** Vitamin C supplementation, pregnancy, hospitalization rate, Ugandan women

## Abstract

**Background:**

Vitamin C or ascorbic acid is a hydro-soluble lactone (synthesized from glucose) essential to human body and available from diet. Despite its broad availability in fruits and vegetables, in many developing countries the incidence of clinical symptoms due to the vitamin deficiency is still very high. Also, pregnant women in the developing countries are frequently hospitalized for several preventable reasons such as anemia in pregnancy, mostly iron-deficient anemia (IDA) and the upper/lower respiratory tract infections (RTI). The aim of the study was to investigate, in a Ugandan rural pregnant women cohort, the preventive effects of vitamin C supplementation on hospital admission.

**Methods:**

384 pregnant women met the inclusion criteria and were randomly assigned to receive either 400 mg of vitamin C daily (187) or not (197) in addition to their standard antenatal vitamins until delivery. The primary outcome measure of this study was to assess the prevention of hospitalization during pregnancy in the group of women supplemented with vitamin C compared to the controls. Fisher‘s exact test was employed in this assessment.

**Results:**

42.2% women in the vitamin C group and 27.9% in the control group were not hospitalized during pregnancy; this difference was found statistically significant.

**Conclusion:**

The results of this study suggest including vitamin C in the guidelines of multivitamin prevention for pregnant women, especially in developing countries where seasonal availability of fruits and vegetables could result in adverse clinical outcomes.

## Background

Vitamin C or ascorbic acid is a hydro-soluble lactone (synthesized from glucose) essential to human body for several functions. Chemically is considered an electron donor (reducing agent or antioxidant), and probably all of its biochemical and molecular roles can be accounted for by this function [1]. Unlike many other animal species, humans and primates lack the terminal enzyme in the biosynthetic pathway for ascorbic acid synthesis, so diet is crucial for its availability in these organisms [2]. Vitamin C is noted for its hydroxylation of lysine and proline residues in the collagen protein of connective tissue and joints. Other important biological functions include copper and iron reduction (which facilitates iron absorption), carnitine biosynthesis, neurotransmitter biosynthesis, reductive protection of folic acid and reductive regeneration of vitamin E [3]. The most important disorder of vitamin C deficiency is scurvy, characterized by gingival changes, pain in the extremities, deformed bone growth in infants, hemorrhagic manifestations, ulcerations and ultimately death [1].

Kallner et al., have demonstrated that at tissue saturation, whole body vitamin C content is approximately 20mg/kg, or 1500mg, and that during depletion its loss is at a rate of 3% of whole-body content per day [4]. Clinical signs of scurvy appear in men at intakes lower than 10mg/day or when the whole-body content falls below 300mg [5]. However, plasma concentrations fall to around 11µmol/l even when dietary vitamin C is between 10 and 20mg/day [6]. Ascorbate is found in many fruits and vegetables. Citrus fruits and juices are particularly rich sources of vitamin C. Brussels sprouts are also rich sources [7]. Despite this wide range availability, in many developing countries the incidence of vitamin deficiency in the population is still very high as its supply is often determined by seasonal factors (availability of water, time and the short harvesting season of many fruits) [8]. Also, a common characteristic of vitamins is their fragility to several environmental factors, so the loss of vitamin on boiling milk can often provide example of a causal infantile scurvy. Natural and synthetic L-ascorbic acid are chemically identical and there are no known differences in their biological activity or bioavailability [9]. Presently, the internationally validated recommended vitamin C intake ranges between 15-25mg/day in children 0-6 months to be gradually raised with age. The adult man and non-pregnant or lactating woman is between 6090mg/day, to add about 10-25mg/day to this value when a pregnant or lactating woman is considered [10-12]. Intakes of 2-3g/day of vitamin C produce unpleasant diarrhea from the osmotic effects of the unabsorbed vitamin in the intestinal lumen in most people [13]. Gastrointestinal disturbances can occur after ingestion of as little as 1g because approximately half of this amount would not be absorbed at this dose [14]. On the other hand, oxalate is an end product of ascorbate catabolism and plays an important role in kidney stone formation, so excessive daily amounts of vitamin C such as intakes higher than 1g/day are shown to produce hyperoxaluria [1].

Since the publication of the book “Vitamin C and the Common Cold” in 1970, by the two times unshared Nobel Prize winner Linus Pauling, a greater interest has been focused on the effects of high dose vitamin intake on the human body [15]. The book highlights the benefit in decreasing the incidence of common cold by a continuous and mega-dose vitamin C intake (>1g/day). Several randomized placebo controlled studies have attempted to repeat Pauling’s experience with inconsistent and variable results which to date can not provide a definite conclusion around the real extent of overall health benefit brought in by vitamin C high daily intake [16-18].

In the developing countries, pregnant women are frequently hospitalized for several preventable reasons such as anemia in pregnancy, mostly iron-deficient anemia (IDA) and respiratory tract infections (RTI) [19]. It is estimated that 33 to 75% of women in developing countries are affected by anemia in pregnancy and its predisposing factors include multi-parity, low socio-economic status, malaria infestation, HIV infection and others [20-21]. Coming to RTI, it is often due to poor housing and clothing also low socio-economic status, poor immune system, malnutrition, close contacts with children under age 3 and “last but not least” improper antibiotic use [19]. It is crucial during the antenatal visit for the physician to assess all relevant factors which could subsequently hamper the optimal child bearing and intervene where necessary to limit all negative future consequences to the mother and phoetus. Unfortunately, in an average primary care or hospital setting in a developing country, in most of the cases this is not possible due to shortcomings of basic laboratory investigation means, drugs, vitamin supplementation availability and adequately skilled nursing staff. As a result of a vicious circle, an average 30-50% of pregnant women do not refer to any health centre unless symptoms are present [22].

All this considerations led us to investigate, in an open-label cohort randomized study, the preventive effects of regular vitamin C supplementation on overall hospitalization rate in the pregnant women population attending a Ugandan rural health centre for antenatal assessment.

The primary outcome measure of this study was to assess the prevention of hospitalization during pregnancy in the group of women supplemented with vitamin C compared to the controls, and the secondary outcome measures were to compare the overall mother-to-child health benefits in the two groups, such as: overall hospitalization rate, weight gain during pregnancy (normal <16kg), term pregnancy (≥37 gestational weeks), preterm delivery, miscarriage (<24 gestational weeks) and child low birth weight (<2500g) and gestational systolic blood pressure.

## Methods

Between August 2007 and January 2009, 400 pregnant women attending the St. Mary’s Health Centre in Kyeibuza (Kiruhura District, southwest of Uganda) for their first trimester antenatal visit (4 to 12 gestational weeks) met the inclusion criteria and were enrolled consecutively in the present study after providing written informed consent. Inclusion criteria were: diagnosis of pregnancy confirmed serologically by B-HCG reagent test along with referred last menstrual period (LMP) not exceeding the past three months, aged at least 18. Exclusion criteria were: referred pregnancy of more than three months by LMP, concomitant HIV infection status, active or recent (< two weeks) sexually transmitted disease (STD) infection, medical record of any severe organ disease such as heart, liver or renal failure at the time of assessment, diagnosis of pregnancy during inpatient admission for any other reason, recent history of multivitamin supplementation (<12 weeks) for any reason, except for pregnancy, and patients incapable to read and write. The Ethics Committee approval was provided by the Local District Health Authority in Kiruhura, and study was conducted according to the principles of Good Clinical Practice (GDP).

Treatment consisted of a chewable tablet and this was the synthetic form of L-ascorbic acid or vitamin C. Each chewable tablet was of 100mg and patients were prescribed two tablets two times a day (2×2) and advised to take with a full glass of water (200ml) regardless of meals but with at least eight hours distance between the intakes. All patients were prescribed daily: ferrous sulphate 200mg, folic acid 5mg and vitamin B-complex 60mg once daily tablets. Tablets were calculated on a four-week basis and repeated at each antenatal visit. The vitamins were provided by Ranbaxy Laboratories Ltd, Gurgaon-India. The study was kindly funded by two Italian humanitarian fund raising organizations: “*Tu ed io insieme*” and “*Oltre le parole*”.

As patients presented to the health centre, those who met the inclusion criteria were randomly assigned to receive either 400 mg of vitamin C daily or not in addition to their standard antenatal vitamins. Supplementation period included complete gestation until delivery. Randomization was obtained by a computer-generated, block design sequence to receive vitamin C or not in a 1:1 ratio. At the same visit, each patient underwent HIV status screening test and RPR-test for syphilis according to the Ugandan Clinical Guidelines (UCG) Recommendations. Patients were reviewed every month until delivery (average 6-8 months) and each of this visits included midwife routine gestational assessment, weight, blood pressure, heart rate and any other obvious body abnormality or gestant complaint. Also, nutritional counseling was provided to all women. Body Mass Index (kg/m²) (BMI) was assessed at study entry and last antenatal before delivery. Though it did not occur, vitamin withdrawal was guaranteed if patients manifested any adverse effect suspected to be related to supplementation.

By reviewing the clinical records of the past five years involving pregnant women’s all cause hospitalization in this rural cohort, we found out that as easily suspected, about 87% of women were admitted to the health centre at least once during pregnancy due to IDA, of which 32% up to two times, 25% up to three times and 11% up to four times. Also, another important comorbidity among the pregnant has been the RTI which involved about 59% of patients together with IDA. Of these, about 48% were admitted twice for different or recurrent infections. Other minor causes had been acute gastroenteritis, threatened abortion and heart palpitations.

We hypothesized to record at least 30% of women not hospitalized during pregnancy in the control group in consideration of the fact they were supplemented regularly throughout their pregnancy with ferrous, folic acid and B-complex for anemia prevention, also followed-up every month, on the other side at least a 50% of women not hospitalized in the group of women supplemented with vitamin C in a 1:1 randomized ratio. Based on these assumptions, at least 200 patients were required per group to detect this difference at a 5% level of significance with 95% power. All patients who met the inclusion criteria and were effectively followed-up were included in the analyses.

Fisher’s exact test was employed to compare the vitamin C group vs. controls with respect to: need or not for hospitalization, term pregnancy, preterm delivery, miscarriage and child low birth weight. The Chi-squared test was used to stratify the two groups in relation to number of times hospitalized during pregnancy. The Student’s independent t-test was used to compare the groups at baseline in relation to age, parity and BMI, also to compare child birth weight at delivery (BW), whereas the paired t-test was used to assess weight gain during pregnancy (WDP) in the two groups of women. Correlation between hospitalization rate and last antenatal BMI was assessed by Spearman’s rank correlation test for each group. Wilcoxon signed rank test was used to assess changes from baseline to delivery in blood systolic pressure (SBP). The Mann Whitney U test was employed to compare average SBP changes in the two groups of women. Analyses were undertaken on per-protocol basis. Statistical calculations were made using GraphPad InStat software.V2.05. Numerical results are expressed as the median and inter quartile range. All tests were two-tailed and significance was reported at the 5% level.

## Results

**Figure 1:**
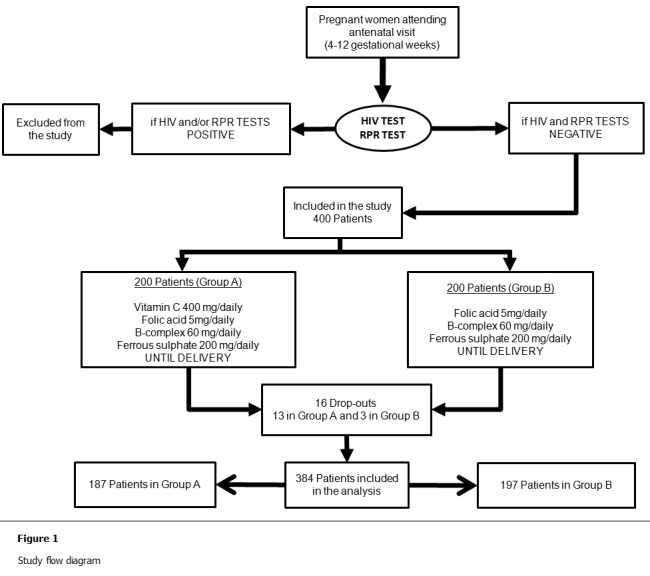
Study flow diagram

Of the four hundred patients assessed and entered into the trial, sixteen were excluded from further analysis after randomization, thirteen in the vitamin C group and three in the control group. Of these, six patients were found to have discontinued their treatment shortly after review and ten patients (7 vs. 3, respectively) failed to come back the following month for review despite repeated attempts to make contact with them. The study flow is presented on Figure 1.

Three hundred and eighty four evaluable patients of which (n=187) in the vitamin C group and  (n=197) in the control group were included in the analysis. There were no significant differences between the two groups at baseline in relation to age, parity and BMI, (Table 1). During pregnancy, 79 out of 187 (42.2%) in the vitamin C group and 55 out of 197 (27.9%) in the control group were not hospitalized and this difference was considered statistically significant. Term pregnancy was achieved by 143 out of 187 women in the vitamin C group and 152 out of 197 in the control group, the difference was considered not significant. For the preterm delivery, 15 vs.18 in the vitamin C group and in the control group respectively were recorded and no statistical difference was found. 29 women in the vitamin C group and 27 in the control group experienced miscarriage, there was no statistical significance found.

**Figure 2:**
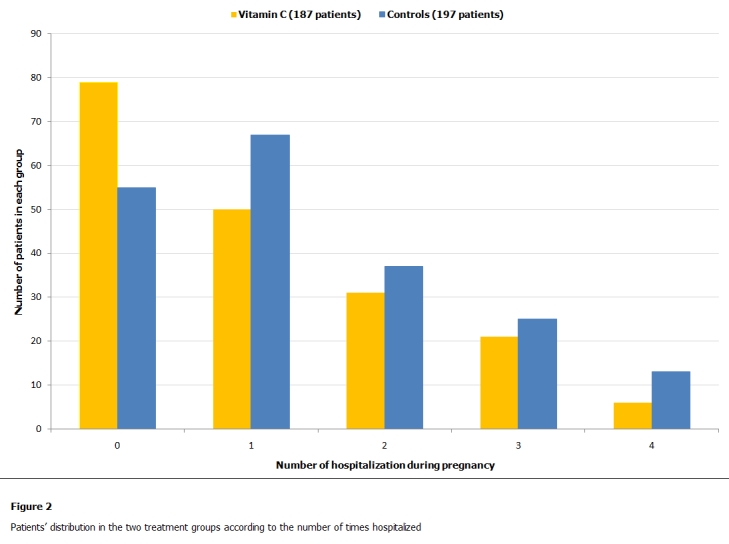
Patients’ distribution in the two treatment groups according to the number of times hospitalized

Child low birth weight was found in 29 women in the vitamin C group and in 47 women among the control group, this difference was considered significant ( Results are summarized in Table 2). Comparing the groups in terms of overall number of hospitalizations during pregnancy, this different trend was found significant, (Table 3 and Figure 2). Average weight gain during pregnancy was of 14kg in the vitamin C group of women and 15 kg in the control group, this difference was found statistically not significant (Table 1). Seemingly, the average child birth weight at delivery was similar in both groups of women and the difference found statistically not significant (Table 1). From baseline to delivery there was a significant increase in systolic blood pressure both in the vitamin C and control groups, (Table 4). When comparing this rise in systolic blood pressure obtained by the two groups, a trend towards significance was found (p=0.059). Seemingly, in both groups a linear correlation was found between the hospitalization rate and the delivery Body Mass Index (vitamin C group (r=0.59), CL 0.49-0.68; control group (r=0.43), CL 0.3-0.54; P=0.0001).

**Table 1:** Patients‘ characteristics and child birth weight

	**Vitamin C**	**Controls**	P*
Age (years) (median, range)	24 (18-39)	25 (18-37)	ns
Parity (children) (range)	4 (0-9)	4 (0-8)	ns
BMI (kg/m²) (median, range)	25 (19-27)	24 (20-27)	ns
WDP (kg) (median, range)	14 (6-27)	15 (4-25)	ns
BW (kg) (median, range)	3.4 (2.79-4.2)	2.99 (2.71-3.99)	ns

* P<0.05 considered significant; ns, not significant; BMI, Body Mass Index; WDP, weight increase during pregnancy; BW, child birth weight

**Table 2:** Numbers of patients (%) in each group after treatment

	**Vitamin C pts)**	**(187 Controls (197 pts)**	**P**	**OR (95% CL)**
Non-hospitalized	79 (42.2%)	55 (27.9%)	0.003	1.89 (1.23-2.89)
Term pregnancy	143 (76.5%)	152 (77.1%)	0.904	0.96 (0.59-1.54)
Preterm pregnancy	15 (8%)	18 (9.1%)	0.719	0.86 (0.42-1.77)
Miscarriage	29 (15.5%)	27 (13.7%)	0.665	1.15 (0.65-2.03)
LBW	29 (15.5%)	47 (23.8%)	0.041	0.58 (0.35-0.97)

Pts: patients, OR: odds ratio, CL: confidence limit, LBW: low birth weight. All P values are given by Fisher‘s exact test.

**Table 3:** Number of patients (%) in each group after stratifying for hospitalization

**Number of hospitalizations during pregnancy**	**Vitamin C (187 pts)**	**Controls (197 pts)**	**P**
0	79 (42.2%)	55 27.9%)	
1	50 (26.7%)	67 (34%)	
2	31 (16.5%)	37 (18.8%)	0.04*
3	21 (11.2%)	25 (12.6%)	
4	6 (3.2%)	13 (6.5%)	

* P value is given by the Chi squared test for independence. Chi squared value=9.971 with 4 degrees of freedom

**Table 4:** Systolic blood pressure (BP) variation from baseline to delivery in the two groups of women

	**Baseline (median, range)**	Delivery* (median, range)	**Average increase (mmHg)**	**P**
Vitamin C	97 (86-134)	104 (92-132)	8.04	0.0001
Controls	98 (83-133)	103 (79-129)	4.5	0.0001

* in cases of miscarriage or preterm delivery, the last antenatal BP value was considered in the analysis

## Discussion

In this randomized, open label, cohort study, the daily vitamin C supplementation provided important clinical signs in the group of women treated. First of all, the rate of women that did not require hospitalization during pregnancy was found significantly higher compared to the group of women undergoing only routine prevention of anemia with iron, folic acid and B-complex supplements. As a confirmation of this data, even when women in the two groups were stratified for overall number of times hospitalized, the vitamin C group was shown to achieve significantly better results.

A high percentage of term pregnancies were achieved in both groups and this could be interpreted as more related to the continuous follow up and regular multivitamin supplementation provided to all women than a real picture of the cohort’s pregnancy outcome. Luckily, few preterm deliveries were encountered in both groups of women, confirming once more the importance of a continuous vitamin supplementation throughout pregnancy. Of all cases (33) only three delivered in our premises ranging from 33 to 35 weeks, all children are followed up and to date enjoy good health, other more serious cases (≤32 weeks) were always rushed to the nearest referral center due to unavailability of neonatal incubators in our setting. Though uncommon, miscarriages where found in both groups and there was not any statistical difference among groups, suggesting absence of any sample bias in the randomization.

In the case of children born with a body weight lower than 2500g, the significant difference found among groups in favor of the vitamin C women is intriguing as it can be interpreted as more and better energy supply to child during gestation. Though a clear explanation of this process is still not available, a hypothesis could be the reduced mother-to-child tissue oxidative stress delivered by the vitamin C regular and high intake. However, when the weight of children born normal was compared among the two groups of women treated, no significant difference was found that could reinforce our assumptions made when considering only those born underweight. Interestingly, although the average weight gain was normal in the two groups during pregnancy without any significant statistical difference, this was found lower in the vitamin C group compared to controls, and this could suggest the possibility of a better fatty acid catabolism provided by high dose vitamin C intake which is known to enhance carnitine synthesis, a fundamental long chain fatty acid transporter to the mitochondrial site for energy production in form of ATP, also, ascorbic acid is involved in the biosynthetic pathway of thyroid hormones which are known regulators of the basal metabolism.

Seemingly, the clinical observation of the characteristic trait of women hospitalized led us to perform a correlation test and a linear correlation was found in both groups between hospitalization rate and the Body Mass Index, which reflects women’s body fitness during child bearing. A strongly important information provided by this analysis is that as woman’s body structure moves from conception to delivery towards overweight or frank obesity, this increases the likelihood of hospitalization for anemia and/or respiratory infections. Possible explanations could be the poor body resistance to physical stress which ends up in reduced red blood cells half-life, also, increased gastric acid secretion, typical in overweight and obese people [23], could limit absorption and thus bioavailability of iron and folic acid all resulting in anemia. On the other hand, the increased weight influences the degree of upper and lower airways opening due to mass effect and therefore limit oxygen exchange which ultimately favor airway microbial overgrowth and invasion in a hygienically poor household. Considering the importance of systolic blood pressure during pregnancy which is a direct reflex of mother’s blood output and therefore child nutrient provision, we compared in each group the difference from baseline to end of pregnancy and a significant average increase was found in both groups from baseline, with a higher increase in the vitamin C group.

This data suggest at least two considerations; first of all regular iron, folic acid and vitamin B-complex protected women from blood and energy loss by stimulating red blood cells and therefore hemoglobin formation. In second place , the higher increase achieved with the vitamin C supplementation (8mmHg vs. 4.5mmHg), with a trend towards significance, can be interpreted in two ways; as an increased gastric absorption of iron in the presence of ascorbic acid that has been demonstrated in several studies [1,3,9,22] and also, the red blood cell protection from oxidation in the presence of high amounts of ascorbic acid which has been demonstrated to be an electron donor of glutathione, a vital antioxidant of red blood cells [1,3,24].

Coming to methods, we intended by recruiting only women in the first trimester to strengthen the validity of our findings, thus shuffled patient supplementation would have made extremely difficult the interpretation of data. The 1:1 randomization scheme was aimed at avoiding cluster errors among women presenting to the health center and thus mimicking a case-control study. The choice of dosing the vitamin C supplement 400mg daily matured after reviewing the medical literature were it was highlighted that at doses exceeding 1g daily the probability of side-effects such as diarrhea and renal colic increased greatly [1,3,13,14] on the other side doses up to 500 mg daily are advised by the official statement of the Linus Pauling’s Institute to the general population, this led us to choose this dose as the safest. That not withstanding, patients were explained to suspend tablets if after less than a week a sudden diarrheal attack was experienced or any difficulty in passing urine not previously present. Alongside, to limit the chances of poor compliance, patients were advised to take all once a day vitamins together far from meals, while distribute the ascorbic acid tablets in order to accompany one dose to other vitamins and another taken alone with at least an eight hour distance, this was explained patiently by the nursing staff in oral and written form.

The strengths of our study were its capacity to show that in a rural Ugandan cohort setting, where sophisticated clinical assessment is not available, the simple regular vitamin intake and a close follow up from diagnosis of pregnancy to delivery, was able to decrease to an important extent the likelihood of patient hospital admittance during the gestational period when compared to the records of the previous five-year pregnancy outcome in the same cohort of women with more or less same clinical and environmental characteristics and in some cases the same women during previous pregnancies. Moreover, the supplementation of a relatively cheap, clinically safe and health-bearing integrator as vitamin C, proved to add overall benefits in terms of hospitalization and several other mother-to-child outcomes relevant during pregnancy. Although we did not achieve the expected 50% of patients not hospitalized in the vitamin C group, the difference between the vitamin C group and controls, 42% vs. 28% was found significant, also is to note the higher amount of drop-outs in the vitamin C group which might have influenced the results (13 vs. 3). We are aware that our results are hampered by the socio-environmental challenges in this cohort of women that ultimately underestimate or in some cases overestimate our findings. Starting from its malaria falciparum endemicity which obviously influence the hospitalization rate regardless of other nutritional factors; then multi parity, in fact, the pregnant women already had an average four children which favored the probability of respiratory tract infections and increased physical stress in family management; the poor socio-economic status which usually do not afford patients a good nutrition and to purchase the regular vitamins throughout their pregnancy, in this study the vitamins were granted free of charge; unavailability of traveling means and good roads to regularly present for follow-up; last but not least is the population stigma for bitter fruits, though available in the land, such as: lemons, oranges and grapefruits which together form one of the richest sources of ascorbic acid.

Despite all positive findings, there are several limitations in this study. Starting from diagnosing the presence or absence of anemia (hemoglobin<10g/dl), which was always done clinically (blood pressure lower than 80/50mmHg, pale conjunctiva and finger tips, asthenia, headache) due to unavailability of any biochemical assay analyzer; no X-ray was performed to understand the exact degree of lung involvement in the respiratory infections, all were assessed clinically (cough, sore throat, hoarseness, chest pain, dyspnea, fever) and treated empirically. Likewise plasma concentration of ascorbic acid was not possible to measure in order to confirm any real difference between women supplemented and not. Lack of appropriate stratification for the precise cause of hospitalization, though the majority were considered as for iron deficient anemia and respiratory tract infections. Among the first, malaria was considered the most important reason for anemia, but it is also true that this was the only objective diagnosis which could be obtained by means of blood smear microscopy. For the respiratory tract, viral were differentiated from bacterial infections based on the degree of temperature rise along with the severity of other breathing and coughing symptoms, nonetheless the clinical response to specific treatments. However, three women in the vitamin C group and four in the control group were admitted for acute gastroenteritis deemed as bacterial then treated with antibiotics and parenteral fluids.

## Conclusion

The regular supplementation of ascorbic acid to pregnant women proved to reduce hospitalization rate during pregnancy and provide overall mother-to-child health benefit, also to be safe as no patient complained or withdrew from the study due to side effects. Our results indicate the benefit of adding vitamin C in the guidelines of multivitamin supplementation to pregnant women. This is particularly important in developing countries were the hazards to a successful pregnancy outcome are greater due to numerous socio-political and environmental obstacles, nonetheless poor nutritional availability of regular fruit and vegetable portions, thus limiting strongly the normal bioavailability of several important vitamins such as ascorbic acid. However, further controlled studies with a larger women population are welcomed in order to confirm the findings of the present study.

## Competing interests

The authors of the present study declare no conflict of interests. The vitamins were provided by Ranbaxy Laboratories Ltd, Gurgaon-India. The study was funded by two Italian humanitarian fund raising organizations: “*Tu ed io insieme*” and “Oltre le parole”.

## Authors‘ contribution

Both doctors, Unim Hans and Byamukama Edward participated in conceiving, preparing and performing the present study alongside with the assistance of their clinical staff.

## Acknowledgements

We are grateful to the fund raising associations “Tu ed io insieme” and “Oltre le parole” for their financial support, to the people and health center staff of Kyeibuza for kindly accepting to participate, Fr. Paolino Tomaino for his moral support, to Mr. Sanjeev Dani of Ranbaxy Laboratories Limited for provision of the vitamin tablets and Prof. Paoluzi for study review and precious advices.
